# Cesarean section as a risk factor for recurrent endometriosis

**DOI:** 10.1016/j.xagr.2026.100707

**Published:** 2026-10-14

**Authors:** George Qiaoqi Chen, Lin Wang, Yunhui Tang, Ying Gu, Qi Chen, Min Zhao

**Affiliations:** 1 Department of Medicine, The Alfred Hospital, Melbourne, Australia (Chen); 2 Department of Gynaecology, Wuxi Maternity and Child Health Care Hospital, Jiangnan University, Wuxi, China (Wang, Zhao); 3 Department of Obstetrics and Gynaecology, Faculty of Medical and Health Sciences, The University of Auckland, Auckland, New Zealand (Wang, Chen); 4 Department of Family Planning, The Obstetrics & Gynaecology Hospital of Fudan University, Shanghai, China (Tang); 5 Department of Obstetrics, Wuxi Maternity and Child Health Care Hospital, Jiangnan University, Wuxi, China (Gu)

**Keywords:** cesarean section, endometriosis, parity, recurrence

## Abstract

**BACKGROUND:**

Endometriosis is a chronic inflammatory disease, with recurrence rates of 20% to 50% within 5 years after surgery. Cesarean section involves tissue injury and inflammation that may contribute to the reactivation of endometriotic lesions. Previous studies suggested an association between cesarean sections and the development of endometriosis. However, it remains unclear whether these factors affect recurrence.

**OBJECTIVE:**

In this study, we investigated the association between previous cesarean sections and the recurrence of endometriosis.

**STUDY DESIGN:**

We retrospectively analyzed 1817 women diagnosed with endometriosis at our hospital.

**RESULTS:**

Recurrent endometriosis occurred in 125 women (6.8%). The median time to recurrent endometriosis was 7 years. Among 1297 women with a history of live birth, 526 (41%) had undergone cesarean section(s). Previous cesarean section(s) significantly increased the recurrence of endometriosis by 84% (*P*=.006), compared to vaginal delivery. In contrast, when compared to nulliparous women, a previous vaginal delivery was not associated with developing recurrence (*P*=.3181). Furthermore, the interval to recurrence was significantly longer in patients with a live birth than in those without a live birth (7.8 vs 5.1 years, *P*=.004).

**CONCLUSION:**

Although endometriosis recurrence after surgery is relatively common, our study suggests that previous cesarean sections significantly increase the risk of recurrence.

**AJOG Global Reports at a Glance**

**Why was this study conducted?**

The potential risk factors for recurrence of endometriosis are unclear.

**Key findings**

A previous cesarean section is significantly associated with an increased risk of recurrent endometriosis. However, women with a live birth had a longer interval to recurrent diagnosis

**What does this add to what is known?**

Our study highlights the importance of considering reproductive history in managing endometriosis.

## Introduction

Endometriosis is a chronic inflammatory condition characterized by the presence of endometrial-like tissue outside the uterus, often resulting in pelvic pain, a common clinical symptom. The global incidence of endometriosis is estimated to range from 6% to 10% among women of reproductive age worldwide.^1^ Despite extensive research, the exact cause of endometriosis remains unclear. There is some evidence that indicates cesarean sections significantly increase the risk of developing both general pelvic endometriosis and scar endometriosis later in life.^2^, ^3^, ^4^

Treatment options for endometriosis include hormonal treatment such as oral contraceptives, lifestyle modifications, and surgical interventions aimed at removing endometriotic tissues.^5^ However, the recurrence rate of endometriosis is approximately 20% within 2 years and 40% to 50% within 5 years after surgery.^6^^,^^7^ One recent study further reported that 62% of women with endometriosis required repeat surgical treatment, with a median interval of 2 years.^8^

Known risk factors for endometriosis recurrence include younger age, the severity of previous endometriosis, the size of endometriotic lesions, the type of surgical intervention, and the presence of ovarian cysts at the time of surgery (reviewed in Refs. ^9^^,^^10^). However, long-term post-operative use of oral contraceptives has been shown to reduce the risk of recurrence, particularly among women not planning future pregnancies. Furthermore, subsequent pregnancies following endometriosis surgery may have a protective effect against the recurrence of endometriosis (reviewed in Ref. ^11^). Currently, studies exploring the association between previous cesarean sections or surgical termination and the recurrence of endometriosis remain limited. A recent study with a moderate sample size (*n*=77) reported that women who have had surgery for endometriosis are twice as likely to experience a recurrence of the disease if they give birth by cesarean section.^12^ However, that study exclusively focused on cesarean sections occurring after endometriosis surgery. Whether a cesarean section performed before two separate diagnoses of endometriosis is a risk factor for recurrence remains unclear.

Given that endometriosis is a chronic inflammatory condition, and surgical procedures like cesarean sections and termination curettage can cause uterine scarring, trigger inflammatory responses, and increase the risk of adhesion (reviewed in Ref. ^13^), it is plausible that such procedures may influence the recurrence or progression of endometriosis. It also raises an important question as to whether cesarean sections performed before endometriosis surgery are associated with a higher risk of disease recurrence. Currently, there is limited evidence to suggest that uterine scars specifically contribute to the migration of endometrial tissue outside the uterus, leading to endometriosis. We therefore conducted this retrospective study to primarily investigate whether a prior cesarean section is associated with an increased risk of recurrence. The secondary aim of this study was to explore whether the timing of a previous cesarean section is also associated with an increased risk of recurrence.

## Methods

This retrospective study was approved by the Ethics Committee of Wuxi Maternity and Child Health Hospital (reference number: 2025/01/0126/25). This study was performed in accordance with the Declaration of Helsinki. The Ethics Committee of Wuxi Maternity and Child Health Hospital waived the requirement for an individual consent form due to the nature of the retrospective study. There was no public involvement in study design and data interpretation.

### Study population

Women with in-hospital diagnoses of endometriosis who underwent surgical treatment at Wuxi Maternity and Child Health Hospital, China, between January 2021 and April 2024 were identified from the hospital databases. The inclusive criteria of this study were (1) surgically and histologically confirmed endometriosis at the initial surgery and (2) repeat surgery for suspected recurrent endometriosis during the study period. The exclusive criteria were (1) endometriosis managed medically, (2) no repeat surgery for suspected recurrence, (3) absence of histological confirmation, and (4) incomplete clinical records. After applying these criteria, a total of 1817 women with surgically and histologically confirmed endometriosis were included in the final analysis.

Diagnosis of endometriosis was based on clinical assessments and imaging findings, including pelvic examinations, ultrasound, magnetic resonance imaging, and/or laparoscopic visualization of lesions. All women included in this study had surgically and histologically confirmed endometriosis, with evidence of endometrial glands, endometrial stroma, or hemosiderin-laden macrophages in tissue samples obtained during surgery.

Recurrent endometriosis was defined as the reappearance of endometriosis-related symptoms, such as pelvic pain with a visual analogue scale score ≥7, in women with a history of surgically and histologically confirmed endometriosis. In women who underwent repeat surgery, the recurrence was confirmed by laparoscopic visualization of endometriotic lesions and subsequent histological examination of excised tissues. Women managed medically or without histologic evidence of recurrent endometriosis were excluded from this study. All women with recurrent endometriosis had been previously treated with laparoscopy with histologically confirmed endometriosis at the initial diagnosis. None received postoperative hormonal or medical treatment following the initial surgery. Recurrent endometriosis was suspected based on clinical symptoms and subsequently confirmed by histological examination after repeat surgical treatment.

Additionally, women with recurrence who had a previous cesarean section were further stratified into the subgroups according to their pregnancy history between the initial diagnosis of endometriosis and the diagnosis of recurrence. (1) Women without a live birth during the interval between the two diagnoses, and (2) women with at least one live birth during the interval between the two diagnoses. The mode of delivery in women who had a live birth was then collected and analyzed.

Data on age at diagnosis, pregnancy history (including parity, surgical terminations, and delivery modes), history of live births, subtypes of prior endometriosis diagnosis, interval between two endometriosis diagnoses, interval between cesarean section and recurrence, and medical history were collected from the hospital’s electronic database. Surgical terminations refer to an abortion performed using dilation and curettage.

### Statistical analysis

Data were presented as mean and standard deviation or as number and percentage, where appropriate. *T*-tests (Mann–Whitney test) or chi-square tests for odds ratio and interquartile range (IQR) were performed for statistical analysis using Prism GraphPad (version 30.0). *P*<.05 was considered statistically significant. Additionally, upper and lower confidence intervals (CIs) were calculated using OpenEpi (version 3.01).

## Results

Of 1817 patients, 618 (34%) were diagnosed with endometriomas, 1099 (60%) were diagnosed with superficial peritoneal endometriosis, 82 (5%) were diagnosed with deep infiltrating endometriosis (DIE), and 18 (1%) were diagnosed with other subtypes, such as cervical and vulvar endometriosis. The clinical demographics of the study cohort are summarized in Table 1. The mean age at diagnosis of current endometriosis was 37 (±8) years. A total of 1297 (72%) had at least one live birth before the current endometriosis, of whom 526 (40.5%) had a history of cesarean sections (Figure). Recurrent endometriosis occurred in 125 patients (7%).

**Table 1:** Demographics of the study cohort (*n*=1817)

Age at diagnosis (y, mean/SD)	36.8±8
Parity=0	520 (28%)
Parity ≥1	1297 (72%)
History of surgical termination(s) (*n*, %)	855 (47%)
Recurrent endometriosis (*n*, %)	125 (7%)
History of cesarean sections (*n*, %) in women with live birth(s) (*n*=1297)	526 (40.5%)

**Figure:**
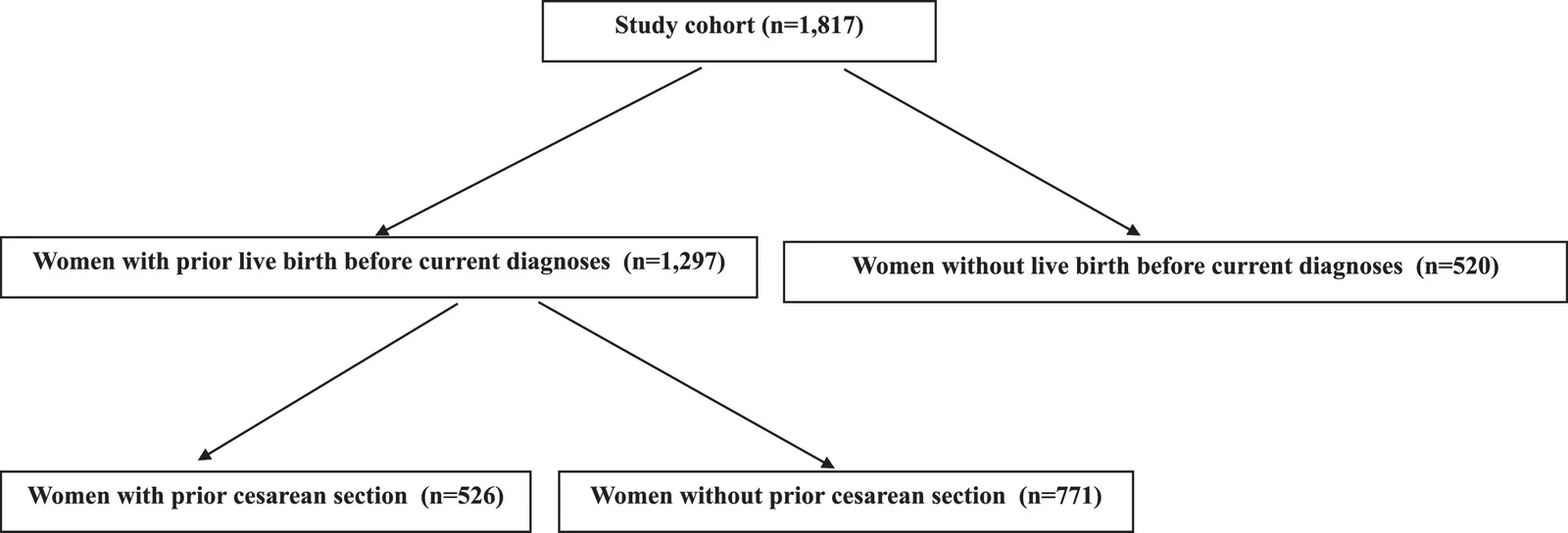
Study cohort and subgroup classification.

Source: Chen. Cesarean section as a risk factor. Am J Obstet Gynecol 2026.

The clinical demographics of women with recurrent endometriosis (*n*=125) and non-recurrent endometriosis (*n*=1692) are summarized in Table 2. The mean age at diagnosis of recurrent endometriosis was 37.7 (±6) years, which was no different from that of non-recurrence. A higher recurrent rate was observed in women with previous endometriomas compared to other subtypes of endometriosis, such as superficial peritoneal endometriosis and DIE (76% vs 24%). Among patients with recurrence, 101 (81%) had a previous live birth, and 55 (54%) of these had a previous cesarean section (Table 2).

**Table 2:** Factors associated with recurrence of endometriosis

	Recurrent endometriosis (*n*=125, 6.8%)	No recurrent endometriosis (*n*=1692, 93.2%)	*P* value
Age at current endometriosis (y, mean/SD)	37.7±6	36.4±6	.076
Previous live birth(s)			
Yes (*n*=1297)	101 (8%)	1196 (92%)	.0179
No (*n*=520)	24 (5%)	496 (95%)	
Previous vaginal delivery			
Yes (*n*=771)	46 (6%)	725 (94%)	0.3181
No live birth (*n*=520)	24 (5%)	496 (95%)	
Previous cesarean section in women with previous live birth(s) (*n*=1297)			
Yes (*n*=526)	55 (11%)	471 (89%)	**.0057 (OR: 1.840, 95% CI: 1.220, 2.749)**
No (*n*=771)	46 (6%)	725 (94%)	
Overall interval to recurrence (y, mean/SD)	7.3±4.6	N/A	N/A
Interval to recurrence in patients with a live birth (*n*=101) (y, mean/SD)	7.8±4.6^a^		
Interval to recurrence in patients without a live birth (*n*=24) (y, mean/SD)	5.1±3.8		
Subtypes of previous endometriosis in women with recurrence (*n*=125)			
Endometriomas (*n*, %)	95 (76%)	N/A	N/A
Others (such as superficial peritoneal endometriosis and DIE) (*n*, %)	30 (24%)		
Previous surgical termination			
Yes (*n*=855)	56 (6.6%)	799 (93.4%)	.3339
No (*n*=962)	69 (7.2%)	893 (92.8%)	

a *P*=.004, compared to patients without live birth. *DIE*, deep infiltrating endometriosis.

We then assessed the association between previous cesarean sections and the risk of developing recurrent endometriosis. The incidence of recurrent endometriosis was significantly higher in patients with a history of cesarean section (*n*=55, 11%) than in those without (*n*=46, 6%; *P*=.0057; Table 2). The odds ratio for developing recurrence in patients with previous cesarean section(s) was 1.840 (95% CI: 1.220, 2.749, *P*<.001). Among women who subsequently developed recurrent endometriosis, the mean interval between the initial and recurrent diagnosis was significantly longer in women with at least one live birth during the interval than that in women without a live birth (7.8±4.6 years vs 5.1±3.8 years, *P*=.004, Table 2). Because live birth was associated with a longer interval between the initial and recurrent diagnoses, we further examined whether the timing of cesarean delivery differed between women with and without recurrence. In 526 women with a cesarean section, the median interval between the last cesarean section and the current diagnosis was 13 years (IQR 8–17) in women with recurrence and 11 years (IQR 7–16.5) in those without recurrence, with no significant difference (*P*=.2304).

To understand whether the timing of cesarean section is also associated with the risk of recurrence, among the 55 patients with recurrence and a history of cesarean section, 41 (74.5%) underwent cesarean section before the initial diagnosis of endometriosis, whereas the remaining 14 (25.5%) underwent cesarean section between the initial diagnosis of endometriosis and the diagnosis of recurrence.

Next, we analyzed the association between previous live birth(s) and the risk of recurrent endometriosis. As shown in Table 2, 101 (8%) patients with previous live birth(s) developed recurrent endometriosis, compared to 24 (5%) patients without previous live birth(s). This difference was statistically significant (Table 2, *P*=.0179). However, when we compared recurrence rates in women with a previous vaginal delivery and those who were nulliparous, there was no statistical difference (Table 2, *P*=.3181). Among those 101 patients with previous live birth(s) and recurrent endometriosis, 72 (71%) did not have a live birth between two diagnoses, while 29 (29%) had a live birth between two diagnoses.

Finally, we analyzed the impact of surgical terminations on the risk of endometriosis. As shown in Table 2, among the 1817 patients, 855 (47%) had a history of surgical termination. In contrast, 962 (53%) patients did not. Women with surgical terminations were older at diagnosis (mean 40 years) compared with those without (mean 34 years) (*P*<.05). The age difference may partially reflect reproductive patterns influenced by China’s previous One-Child Policy, which ended at the end of 2015. Additionally, our recent study^14^ reported a relatively lower awareness of contraceptive use in the Chinese population, with only 41% of women recognizing its importance and 22% reporting no contraceptive use. Taken together, these demographic, policy, and behavioral factors likely contributed to the higher frequency of surgical terminations observed in our cohort.

## Discussion

In this study, we found that the incidence of recurrent endometriosis was 7%, with a mean recurrence interval of 7.3 years. A significantly higher number of women with recurrence had previous cesarean section(s), with an 84% increased risk of recurrence. Live births delay the development of recurrence.

The estimated recurrence rate of endometriosis varies depending on the duration of follow-up and definition of recurrence, ranging from 6% to 67%.^15^^,^^16^ Several risk factors for recurrence have been discussed, including the location of endometriotic lesions, age at menarche, parity, and previous medical treatment of endometriosis (reviewed in Refs.^9^^,^^10^). However, the exact mechanism is still unknown. This is consistent with a recent study showing that an increased risk of developing recurrent endometriosis was observed in women who had a cesarean section after surgical treatment of the initial endometriosis.^12^ Our data confirmed that a previous cesarean section was associated with an 84% increased risk of recurrent endometriosis. However, this finding should be interpreted with caution. As this is a retrospective study without adjustment for potential confounders, the observed association may be influenced by indication bias and may not be solely attributable to cesarean section. Thus, our findings should be considered hypothesis-generating rather than causal.

Additionally, we further found that previous vaginal deliveries were not associated with developing recurrence by comparing with nulliparous women. One possible mechanism of the association between cesarean section and recurrence is the presence of cesarean scar disorder (uterine niche), also known as isthmocele. This condition, characterized by a defect or niche at the cesarean incision site, has been associated with altered menstrual flow dynamics, chronic inflammation, and impaired uterine healing.^17^ These changes may promote retrograde menstruation and create a favorable environment for endometrial cell implantation and survival, thereby contributing to the persistence or recurrence of endometriosis.^18^^,^^19^ In this study, we did not specifically assess cesarean scar disorder. However, we found that among patients with recurrence who had a history of cesarean section (*n*=55), 74.5% did not have a live birth between the two diagnoses. This finding suggests that the increased risk of recurrence associated with cesarean section may be regardless of the timing of the previous cesarean section, potentially indicating a broader influence of cesarean-related uterine changes, including cesarean scar disorder, on disease persistence or recurrence. Further research integrating imaging or surgical evaluation of the uterine scar could help clarify this potential mechanism.

Studies reported that 20% of patients experience recurrence within 2 years, and nearly 50% develop recurrence within 5 years after initial surgical treatment.^6^^,^^7^ Our current study found that 7% of patients experienced recurrence with a mean of 7.3 years, ranging from 1 to 22 years after the last diagnosis. The lower recurrence rate reported in our cohort (7%) is more likely attributable to underestimation due to other factors, including patients with mild clinical symptoms who did not undergo surgical evaluation or seek treatment at other institutions. Additionally, it is well-known that there is often a delay in the diagnosis of endometriosis.^20^ However, our findings suggest that recurrence may occur at any point after surgical intervention, underscoring the need for both short-term and long-term clinical monitoring. Our study reported that only 20% of recurrences occurred in nulliparous women, and current evidence suggests that pregnancy does not prevent endometriosis recurrence. This is because the factors potentially influencing the development of endometriosis are difficult to evaluate.^21^^,^^22^ However, our data show that the interval to recurrence was significantly longer in patients with a live birth than in those without. These findings suggest that the pregnancy experience may delay, but not eliminate, the recurrence of disease. Additionally, our data also show that the increased recurrence risk associated with cesarean section is not related to the timing of the previous cesarean section.

A follow-up case-control study reported that cesarean sections significantly increased the risk of developing endometriosis in women without a prior history of endometriosis within 10 years, with 60% of patients having undergone cesarean sections.^2^ In contrast, our study found that 40% of patients had a history of cesarean sections and 60% of patients had vaginal deliveries. Our data further showed that surgical terminations do not increase the risk of developing endometriosis. The discrepancies between our findings and the study led by Andolf^2^ can be explained that (1) the study was a prospective follow-up study with over 600,000 women, while our study was retrospective; (2) the patient selection in that study used ICD codes to identify endometriosis, whereas in our study, all patients were surgically and histologically confirmed. These differences in study design and patient selection limit direct comparability between the two studies.

Furthermore, a recent study showed no association between cesarean sections and adenomyosis, a condition with clinical symptoms that are similar to endometriosis.^23^

Current evidence suggests that the majority of endometriotic lesions do not regress during or after pregnancy.^24^ In our current study, we found that 8% of women with live births developed recurrence. Among women with recurrence who had a live birth (*n*=101), 29% developed recurrent endometriosis after a live birth between the two diagnoses. This finding further suggests that pregnancy does not necessarily prevent the recurrence of the condition. However, data on reproductive intentions for the remaining 71% of patients are unavailable. Emerging evidence suggests an association between isthmocele (niche) and endometriosis.^18^^,^^19^ Isthmocele results from a cesarean section, and this defect in the uterine scar can be a cause of secondary infertility.^17^ The possible underlying mechanism is that this defect can trap blood and mucus, leading to inflammation, potential sperm transport issues, and impaired embryo implantation.^17^ Given that endometriosis is often associated with secondary infertility, it remains unclear whether the absence of a subsequent pregnancy in 71% of our patients reflects a personal choice or subfertility.

Our findings, which show an association between cesarean section and the risk of endometriosis recurrence, need to be confirmed by future prospective studies, ideally randomized clinical trials. Our current study primarily evaluated the association of recurrent endometriosis in women with surgically and histologically confirmed endometriosis. Our findings also need to be confirmed in those with medical management in the future. Additionally, detailed previous cesarean section, including uterine closure technique, cesarean scar niches, postpartum hemorrhage, associated surgical intervention at cesarean section, and the number of cesarean sections, were unavailable for analysis. These potential confounders in the development of recurrent endometriosis need to be investigated in the future.

Cesarean section would have important public health implications. In women with a history of endometriosis, cesarean section should be considered more judiciously, as it is one of the potential risks of recurrence. Additionally, future studies should examine potential preventive surgical strategies. These studies include incision techniques, peritoneal irrigation, and timing of cesarean section between pregnancies, which can influence the risk of adhesion formation, inflammation, and implantation, which are associated with endometriosis.^13^

In conclusion, although the recurrence of endometriosis is relatively common, our study found that a history of cesarean section was more frequent among women with recurrence. Additionally, a live birth may delay, but not prevent, the recurrence of the condition. Our findings highlight the importance of considering reproductive history in managing endometriosis. Future prospective studies are needed to confirm these findings.

## CRediT authorship contribution statement

**George Qiaoqi Chen:** Writing – review & editing, Writing – original draft, Investigation, Conceptualization. **Lin Wang:** Data curation. **Yunhui Tang:** Conceptualization. **Ying Gu:** Conceptualization. **Qi Chen:** Writing – review & editing, Investigation, Formal analysis, Conceptualization. **Min Zhao:** Writing – original draft, Funding acquisition, Conceptualization.
